# In Vitro/In Vivo Metabolism of Ginsenoside Rg5 in Rat Using Ultra-Performance Liquid Chromatography/Quadrupole-Time-of-Flight Mass Spectrometry

**DOI:** 10.3390/molecules23092113

**Published:** 2018-08-22

**Authors:** Chao Hong, Ping Yang, Shuping Li, Yizhen Guo, Dan Wang, Jianxin Wang

**Affiliations:** 1 Department of Pharmaceutics, School of Pharmacy, Fudan University & Key Laboratory of Smart Drug Delivery, Ministry of Education, Shanghai 201203, China; cpuhongchao@126.com (C.H.); guo.1269@osu.edu (Y.G.); 15111030048@fudan.edu.cn (D.W.); 2 Instrumental Analysis Center, School of Pharmacy, Fudan University, Shanghai 201203, China; 15111030018@fudan.edu.cn; 3 The MOE Key Laboratory for Standardization of Chinese Medicines and The SATCM Key Laboratory for New Resources and Quality Evaluation of Chinese Medicines, Institute of Chinese Materia Medica, Shanghai University of Traditional Chinese Medicine, Shanghai 201210, China; after100hong@126.com; 4 Institute of Materia Medica, Academy of Integrated Chinese and Western Medicine, Fudan University, Shanghai 200040, China

**Keywords:** ginsenoside Rg5, metabolism, in vitro/in vivo, UPLC/QTOF-MS

## Abstract

*Background*: Ginsenoside Rg5 has been proved to have a wide range of pharmacological activities. However, the in vitro and in vivo metabolism pathways of ginsenosides are still unclear, which impedes the understanding of their in vivo fate. In this paper, the possible metabolic process of Rg5 was studied and the metabolites are identified. *Methods*: Samples from rat liver microsomes (RLMs) in vitro and from rat urine, plasma and feces in vivo were collected for analysis after oral administration of Rg5. A rapid analysis technique using ultra-performance liquid chromatography (UPLC)/quadrupole-time-of-flight mass spectrometry (QTOF-MS) was applied for detecting metabolites of Rg5 both in vitro and in vivo. *Results*: A feasible metabolic pathway was proposed and described for ginsenoside Rg5. A total of 17 metabolic products were detected in biological samples, including the RLMs (four), rat urine (two), feces (13) and plasma (four). Fifteen of them have never been reported before. Oxidation, deglycosylation, deoxidation, glucuronidation, demethylation and dehydration were found to be the major metabolic reactions of Rg5. *Conclusions*: The present study utilized a reliable and quick analytical tool to explore the metabolism of Rg5 in rats and provided significant insights into the understanding of the metabolic pathways of Rg5 in vitro and in vivo. The results could be used to not only evaluate the efficacy and safety of Rg5, but also identify potential active drug candidates from the metabolites.

## 1. Introduction

Ginseng is one of the most famous medicinal herbs, and has been used widely in China, Japan and Korea over thousands of years [1,2,3]. Numerous studies have reported that ginseng promotes a wide range of pharmacologic activities in the central nervous, endocrine, cardiovascular and immune systems [4,5,6,7,8,9]. Ginsenosides, as a diverse group of steroidal saponins, are generally believed to be the main bioactive compounds found in ginseng. Recently, it has been reported that ginsenosides have promising therapeutic potential for the treatment of diabetes, Alzheimer’s disease, depression and cancer [10,11,12,13,14,15,16]. Nowadays, accumulating evidence has indicated that the biological and pharmacological activities of steamed ginseng (black and red ginseng) are much greater than those of non-steamed ginseng (white ginseng). This is mainly contributed to the fact that the non-steamed ginseng components could be extensively converted to less polar-degraded ones during the steaming process [17,18].

**Figure 1:**
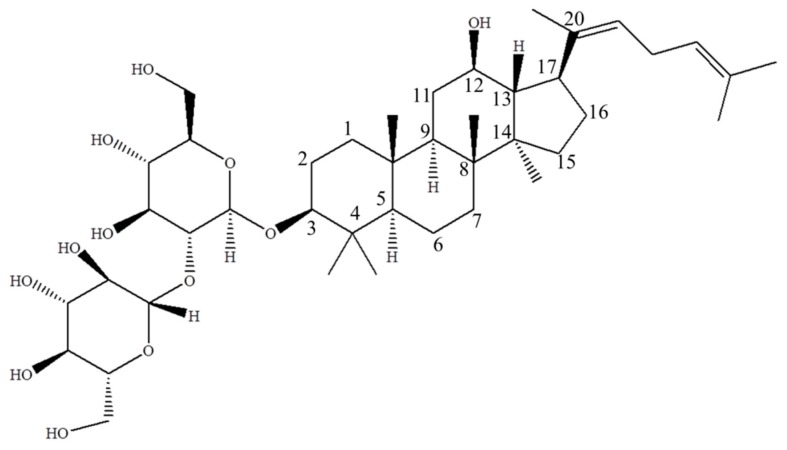
The structure of ginsenoside Rg5 ((3*β*,12*β*,20*E*)-12-hydroxydammara-20(22), 24-dien-3-yl-2-*O*-β-d-glucopyranosyl-β-d-glucopyranoside, MW = 767), a major rare saponin generated during steaming treatment of ginseng.

Ginsenoside Rg5 (Figure 1), a major rare saponin produced during ginseng steaming treatment, has been proved to display various pharmacological effects. A study performed by Kim et al. demonstrated that Rg5 could protect against memory deficit by inhibiting AChE activity and increasing BDNF expression, which was critical to the treatment of Alzheimer’s disease [19]. It was also reported that Rg5 could suppress ROS production by upregulating hemeoxygenase-1 (HO-1) expression in LPS-stimulated BV2 cells, which explains the potential therapeutic effect for various neuroinflammatory disorders [20]. Ginsenoside Rg5 could also suppress oxazolone-induced ear contact dermatitis swelling through reducing the mRNA expressions of cyclooxygenase-2, interleukin 1β, tumor necrosis factor (TNF)-α and interferon (IFN)-γ [21]. In addition, Rg5 was also regarded as a potential cytotoxic or genotoxic drug for the treatment of various kinds of cancers. For example, Rg5 promoted breast cancer cell apoptosis in a multi-path manner with higher potency than ginsenoside Rg3 in MCF-7 and MDA-MB-453 cell lines [22]. Rg5 demonstrated genotoxic effects in the HeLa and MS751 cells by increasing the level of DNA damage [23]. Besides that, Rg5 could downregulate the activity of cyclin dependent kinase, which blocked the cell cycle at G_l_/S transition phase in SK-HEP-1 cancer cells [24]. In summary, increasing attention has been paid to this rare saponin, which has huge potential to be developed into a novel drug for future treatment of cancer, inflammation and Alzheimer’s disease [25,26,27].

Since ginsenosides are easily degraded in the body after oral administration, it is necessary to investigate the metabolism process of ginsenosides to elucidate their efficacy and side effects. Cui et al. determined that less than 2% of orally ingested dose of ginsenosides were recovered in human urine, indicating that most of the ginsenosides could not be absorbed in the original form [28]. Only secondary metabolites of ginsenosides could reach the systemic circulation and display therapeutic effects [29]. Therefore, metabolism experiments on enzymes, intestinal bacteria, acids and animals have been undertaken to find these secondary metabolites with therapeutic effects [30,31,32,33]. For example, in the study performed by Shin et al., ginsenoside Rh2 was confirmed to be one of the metabolites of Rg3, and exhibited more potent antiallergic effects in the body than Rg3, such as anti-inflammatory effects and anti-passive cutaneous anaphylaxis [34]. Therefore, the study of ginsenoside metabolism can help us to better understand the in vivo fate and mechanisms of action of these compounds, which is critical to the clinical development and application of ginsenosides.

In this study, we focused our interest on the in vivo and in vitro metabolism of ginsenoside Rg5. Previous studies on the metabolism of Rg5 were narrowly focused on human intestinal microflora in vitro, where, only one metabolite ginsenoside Rh3 was obtained [35]. Investigations also found that the metabolite Rh3 was more potent than the parent drug Rg5 in inhibiting chronic dermatitis and psoriasis. However, the data was too limited to clearly illustrate the actual metabolism process and therapeutic mechanism of Rg5. In this study, several breakthroughs have been made in this research field: a specific and sensitive method using ultra-performance liquid chromatography/quadrupole-time-of-flight mass spectrometry (UPLC/QTOF-MS) is established for the first time to quickly analyze the possible metabolites of Rg5 both in vitro and in vivo. Seventeen metabolic products of Rg5 were identified in different excreted samples, of which 15 have not been reported before. Among all the metabolites, 13 are found in feces, two in urine, four in plasma and rat liver microsomes (RLMs) respectively. Finally, the metabolism of ginsenoside Rg5 was systematically elucidated.

## 2. Results and Discussion

### 2.1. Mass Spectral Properties of Rg5

**Figure 2:**
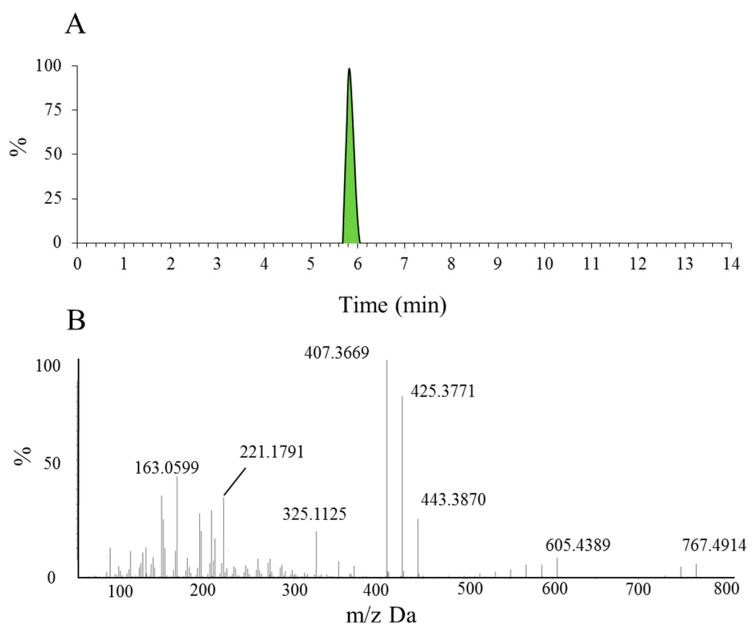
The extracted ion chromatogram of Rg5 (**A**) and the MS/MS spectrum of Rg5 using ESI in positive ion mode (**B**).

**Figure 3:**
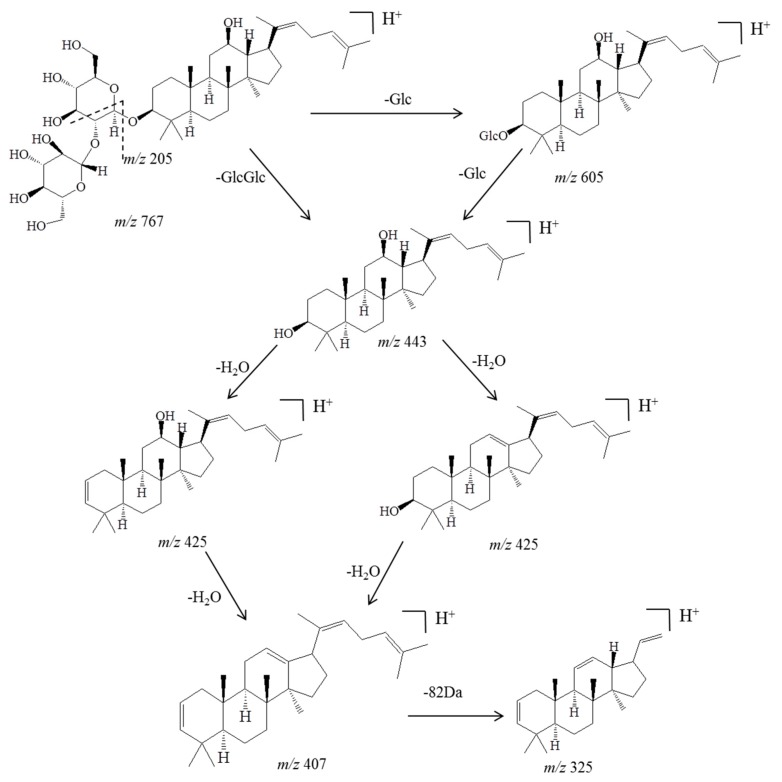
Major MS/MS fragmentations proposed for ginsenoside Rg5, which was crucial for further analysis and identification of the metabolites.

Before identifying the potential metabolites, the mass spectral behavior of the parent compound should be comprehensively investigated and understood. In our preliminary study, positive and negative modes were compared to find the best experimental conditions. Generally, many more product ions with sufficient abundance could be observed in the positive mode than in negative mode, which provided more information for analysis. Thus, positive mode ESI mass spectra were applied in the present study. Furthermore, the source temperature was also tested in order to improve the intensity of the response. When the temperature increased to more than 200 °C, the response of mass spectral of Rg5 and its metabolites dramatically decreased, which was due to the high temperature instability of ginsenosides [36,37,38,39]. Therefore, a final source temperature of 121 °C was chosen to perform the experiments. As Figure 2A shows, Rg5 had a retention time of 5.95 min under these experimental conditions, and showed typical dissociation fragment ions at *m*/*z* 605, 443, 425, 407, 325, 221, 163. The observed fragment ions at *m*/*z* 605 and 443 were attributed to the reduction of one and two glucose molecules at C_4_ position, respectively. The fragment ions at *m*/*z* 425 and 407 observed at high abundance originated from the elimination of one and two H_2_O molecules, respectively. Accordingly, the product ion of *m*/*z* 163 corresponded to a protonated glucose residue ion. Then, further neutral loss of a C_6_H_10_ side chain at the C-20 position could generate an ion at *m*/*z* 325. Finally, a product ion at *m*/*z* 221 was yielded by the cross-ring cleavage of the glucose molecule of the disaccharide moiety. Major MS/MS fragmentations pathways proposed for ginsenoside Rg5 are shown in Figure 3. 

### 2.2. Metabolites of Rg5 in RLMs

**Figure 4:**
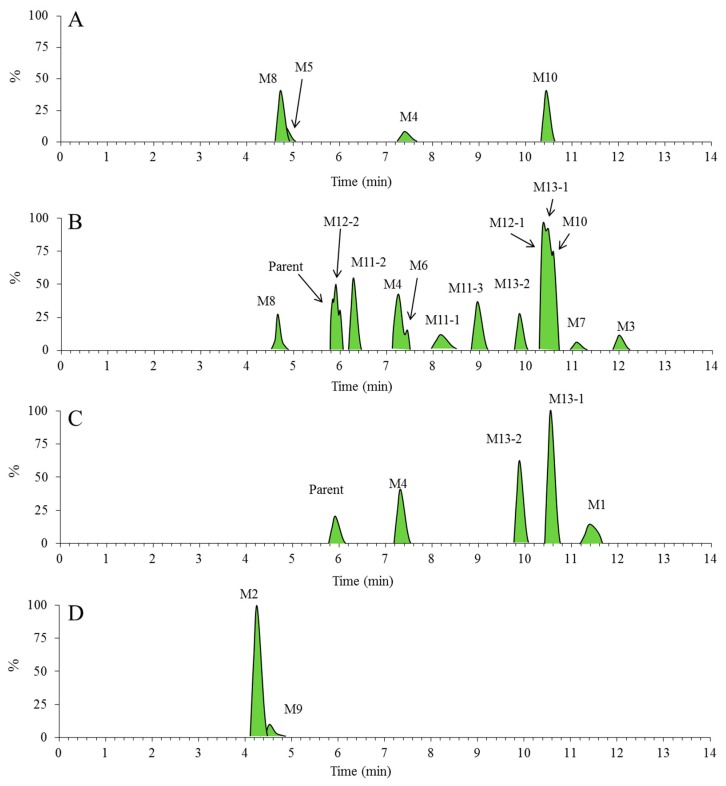
The extracted ion chromatograms for metabolic profile of Rg5. Numbers are labeled for each metabolite sample from RLMs, urine, feces and plasma ((**A**) the RLMs of 1 h; (**B**) the feces of 0–24 h; (**C**) the plasma of 0–4 h; (**D**) the urine of 0–24 h).

After incubating Rg5 with RLMs for 60 min, various metabolites of Rg5 were explored by UPLC/ESI-QTOF-MS. As the extracted ion chromatograms show (Figure 4A), four major metabolites of Rg5 were generated from CYP450 isozymes in RLMs, i.e., M4, M5, M8 and M10.

### 2.3. Metabolites of Rg5 Detected in the Urine, Feces, Plasma Samples of Rats after Oral Administration 

To identify the possible metabolites of Rg5 after oral administration, a full scan mass spectra of fragment ions of three in vivo samples, i.e., urine, feces, and plasma of rats, were compared with those of blank samples. As shown in Figure 4B–D, 15 metabolites of Rg5 (M1 ~ 4 and M6 ~ 13) were observed in vivo (the data of randomized controlled trial was not shown in the text). Except for M2, M5 and M9, most of the metabolites were detected in feces. The metabolite M5 was only detected in vitro. More information about those metabolites is provided in Table 1.

#### 2.3.1. Metabolite M1

**Figure 5:**
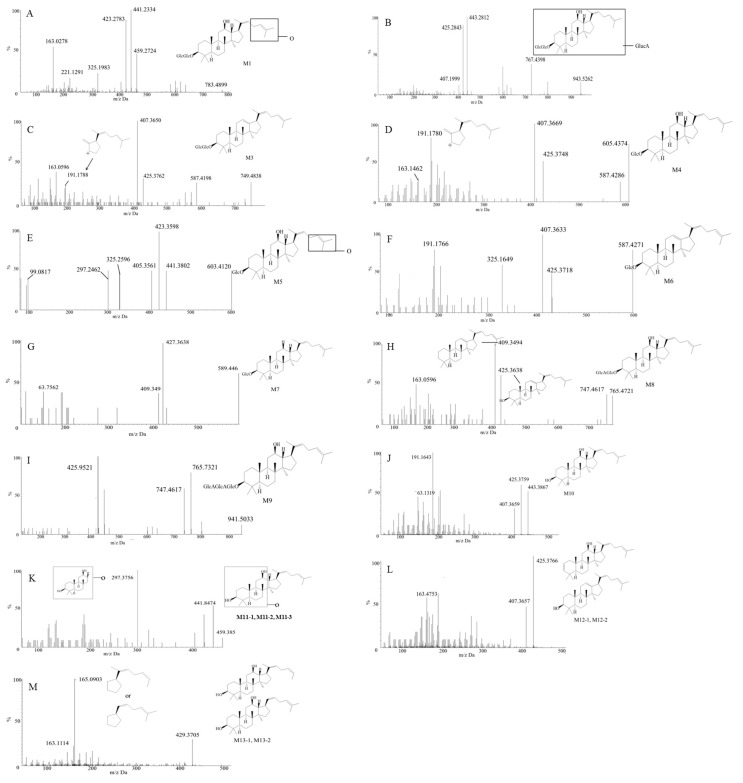
MS spectra of Rg5 metabolites collected from RLMs, urine, feces and plasma on UPLC/QTOF-MS (A–M represents for MS/MS spectrum of the deprotonated metabolite M1–M13).

Figure 5A shows the MS/MS spectrum of the deprotonated metabolite M1, which had a retention time of 11.31 min and showed a [M + H]^+^ ion at *m*/*z* 783, which was 16 Da more than that of parent compound Rg5, indicating that it is a monooxidation metabolite of Rg5. The product ion at *m*/*z* 459, which was 16 Da heavier than the [aglycone + H]^+^ at *m*/*z* 443, could be considered as a monooxide of aglycone. The appearance of a fragment ion at *m*/*z* 325, which was generated from the loss of aliphatic side chain, provided clear evidence that the oxidation also occurred on the side chain. However, the exact oxidation sites of the metabolites were not determinable. Similar to the parent ginsenoside Rg5, the MS/MS spectra of M1 also exhibited deglycosylated and dehydrated product ions, including 459 [M + H − 324]^+^, 441 [M + H − 342]^+^ and 423 [M + H − 360]^+^. In addition, the fragment ions at *m*/*z* 163, 221 and 325 in the MS/MS spectra of M1were also the same as those of the parent compound Rg5. 

#### 2.3.2. Metabolite M2

Metabolite M2 exhibited a [M + H]^+^ ion at *m*/*z* 943, which was 176 Da greater than that of the protonated parent compound. The product ion *m*/*z* 767 [M + H − 176]^+^ was also observed as a single peak in a neutral loss of 176 scan in the MS/MS spectrum (Figure 5B), suggesting the presence of a glucuronic acid unit. In addition, other mass spectral fragmentation peaks, such as 443, 425 and 407, were the same as its parent compound Rg5, indicating that M2 could be tentatively assigned as a glucuronide of Rg5. Furthermore, the much shorter retention time of M2 (4.3 min) compared to the parent compound also validated the notion that M2 is a glucuronide metabolite of Rg5.

#### 2.3.3. Metabolite M3 

Metabolite M3 exhibited a retention time of 12.05 min and a [M + H]^+^ ion at *m*/*z* 749. This was 18 Da less than that of the protonated parent compound, which indicated that it was a dehydration metabolite. The MS/MS spectrum in Figure 5C showed fragment ions at *m*/*z* 587 (−162), 425, 407, and 191. The product ions that appeared at *m*/*z* 587 and 191 were obviously different from the fragmentation behavior of the parent compound. The fragment ion at *m*/*z* 587 was yielded via the loss of a glucose fragment from the molecular ion at *m*/*z* 749. The fragment ion of *m*/*z* 191 indicated the dehydration occurred at C_12_ and C_13_ (Figure 5C). 

#### 2.3.4. Metabolite M4 

Metabolite M4 (retention time t_R_ = 7.38 min), detected both in vitro and in vivo, exhibited a [M + H]^+^ ion at *m*/*z* 605, which was 162 Da lower than that of the parent compound. Comparing with the reference standard, it was identified as Rh3, generated by the elimination of a glucose residue of Rg5. Figure 5D showed the MS/MS spectrum of M4, most of whose product ions were identical to those of Rg5, such as the fragments at *m*/*z* 425, 407, 325 and 163. In addition, the fragment ion at *m*/*z* 587 was generated via the reduction of a glucose and a H_2_O fragment from the molecular ion at *m*/*z* 605. The fragment ion of *m*/*z* 191 was in common with M3.

#### 2.3.5. Metabolite M5 

Metabolite M5 (retention time t_R_ = 4.87 min) was only detected in vitro. It exhibited a [M + H]^+^ ion at *m*/*z* 621, which was 146 Da lower than that of the protonated compound, suggesting the deglycosylation and oxidation of Rg5. The fragment ions showed in MS/MS spectra (Figure 5E) were *m*/*z* 603, 441, 423, 405, 325 and 99. The loss of a glucose from M5 yielded the product ion at *m*/*z* 603. The fragment ion of *m*/*z* 99 was generated via the oxidation of the side chain, indicating that the modification occurred on the side chain moiety. Other fragment ions were in common with those of M1, suggesting M5 was also a deglycosylated metabolite of M1.

#### 2.3.6. Metabolite M6 

Metabolite M6 showed a retention time of 7.38 min and a [M + H]^+^ ion at *m*/*z* 587. The lower *m*/*z* of 180 Da compared with Rg5 indicated the deglycosylation and dehydration of the parent compound Rg5. The MS/MS spectra (Figure 5F) exhibited major fragment ions at 425, 407, 325 and 191, which were the same as the low *m*/*z* sections of M3. Therefore, M6 was assigned as a deglycosylated M3. 

#### 2.3.7. Metabolite M7 

Metabolite M7 with a retention time of 11.2 min showed a [M + H]^+^ ion at *m*/*z* 589, which was 178 Da less than that of the protonated Rg5, indicating the occurrence of deglycosylation and deoxidation at C_3_ and C_12_ respectively. As shown in Figure 5G, the MS/MS spectra demonstrated major fragment ions at 427, 409 and 163. Obviously, the fragment ions at *m*/*z* 427 and 409 did not appear in the protonated product ion series of the original compound Rg5. They were attributed to the loss of a glucose residue and a H_2_O molecule at C_3_ of the molecule at *m*/*z* 589, respectively. As a result, except for deoxidation, other reactions of M7 were identical to M4. Therefore, M7 could be assigned as a deoxidized M4. 

#### 2.3.8. Metabolite M8 

Metabolite M8 showed a retention time of 4.84 min and a [M + H]^+^ ion at *m*/*z* 765, which was 2 Da (−162, +176, −16) lower than that of the protonated parent compound, indicating its deglycosylation, deoxidation and glucuronidation. Figure 5H showed major fragment ions in the MS/MS spectra at 747, 425, 409 and 163. The loss of a H_2_O fragment from the molecular ion at *m*/*z* 765 generated the product ion at *m*/*z* 747, meaning that the hydroxyl groups at C_12_ still existed in the molecule. Therefore, glucuronidation should happen at the position of C_3_. In addition, the structure of the fragment ions at 425 and 409 are shown in the Figure 5H.

#### 2.3.9. Metabolite M9 

Metabolite M9 (retention time t_R_ = 4.3 min) exhibited a [M + H]^+^ ion at *m*/*z* 941, which was 176 Da heavier than that of the M8. Furthermore, the MS/MS spectra in Figure 5I showed major fragment ions at 765, 747 and 425, which were identical to those of M8. Therefore, M9 was deduced as a glucuronide of M8.

#### 2.3.10. Metabolite M10 

As a deglycosylated metabolites of Rg5, M10 (retention time t_R_ = 10.69 min) exhibited the [M + H]^+^ ion at *m*/*z* 443 (Figure 5J). The reduction of 324 Da from the parent drug was consistent with two glucose moieties. Besides, M10 had almost the uniform fragment ions with the low *m*/*z* section of Rg5 and M4. Therefore, M10 was identified authentically as quasiprotopanxadiol (QPT), by comparison with the reference standard.

#### 2.3.11. Metabolite M11 

M11-1, M11-2 and M11-3 (retention time t_R_ = 8.2, 6.33 and 9.01 min, respectively) were three isomers with identical protonated molecular weights of 459. They were 308 (−162, −162, +16) Da lower than that of the protonated parent compound, which was caused by the introduction of an oxygen atom and the reduction of two glucose moieties. The MS/MS spectra (Figure 5K) of them showed some identical fragment ions at *m*/*z* 441 (−18), 423 (−36) and 297. The fragment ions at *m*/*z* 441 and 423 resulted from the loss of one or two H_2_O molecules of M11. The fragment ion at *m*/*z* 297 was not shown in the MS/MS spectra of the parent compound, suggesting that the +16 modification occurred on the ABC ring. Therefore, M11 were identified as oxides of QPT, though the exact positions were hard to distinguish due to limited information.

#### 2.3.12. Metabolite M12 

M12-1and M12-2 were two isomers, which both exhibited a [M + H]^+^ ion at *m*/*z* 425. It was 342 (−162, −162, −18) Da lower compared to that of the protonated parent compound, indicating the reduction of two glucose moieties with dehydration. As Figure 5L shows, the main fragment ions of the MS/MS spectra of M12-1 and M12-2 were quite similar to those of M10, with product ions such as 425, 407 and 163, found in their MS/MS spectra. Consequently, they were identified as dehydration products of M10, which had lost one H_2_O at C_12_ or C_3_. However, it is hard to identify the exact structure of the M12-1 and M12-2, because no more information was available to help identify the position of dehydration. 

#### 2.3.13. Metabolite M13

M13-1 and M13-2 (retention time t_R_ = 10.54 and 9.89 min, respectively) both showed a [M + H]^+^ ion at *m*/*z* 429, which was 342 (−162, −162, −14) Da lower than that of the protonated parent compound, indicating the loss of two glucose moieties with demethylation. Figure 5M provided the MS/MS spectra of M13-1and M13-2, which were quite analogous. The demethylation of the side chain generated the fragment ion at *m*/*z* 165, indicating the −14 modification occurred on the side chain. There were two methyl groups on the side chain. Due to the difference of retention times between M13-1 and M13-2, it was believed that they lost different methyl groups. However, it is hard to distinguish the exact position of demethylation for the M13-1 and M13-2 due to limited data.

### 2.4. Metabolic Pathways of Rg5

Ginsenosides have been supposed to exert pharmacological effects via their secondary metabolites, which was equally important to the original form. Rg5 is one of the ginsenosides with various strong activities. However, the metabolite profile of Rg5 absorbed through digestive tract was unclear, not to say the structures of the metabolites. Therefore, in the present study, the metabolite process and metabolically labile sites of ginsenoside Rg5 in rats is clearly determined for the first time.

A sensitive and specific UPLC/ESI-QTOF-MS technique was developed, and applied to identify the possible metabolites in urine, plasma and feces of rats after oral administration of Rg5. In order to detect the accurate masses and element compositions of the metabolites, HRMS (quadrupole time-of-flight) technology was used. Results showed that 17 metabolites were identified in different excretion samples. Except for Rh3 and QPT, other 15 metabolites have never been reported before. Only very weak signals of parent drug Rg5 could be detected in the feces and plasma samples, but nothing in the urine. More importantly, among the 17 metabolites, 13 metabolites were found in feces, while only two were found in urine and four in plasma and RLMs, respectively. Therefore, faecal clearance was found to be the major excretion route of Rg5 and its metabolites. 

**Figure 6:**
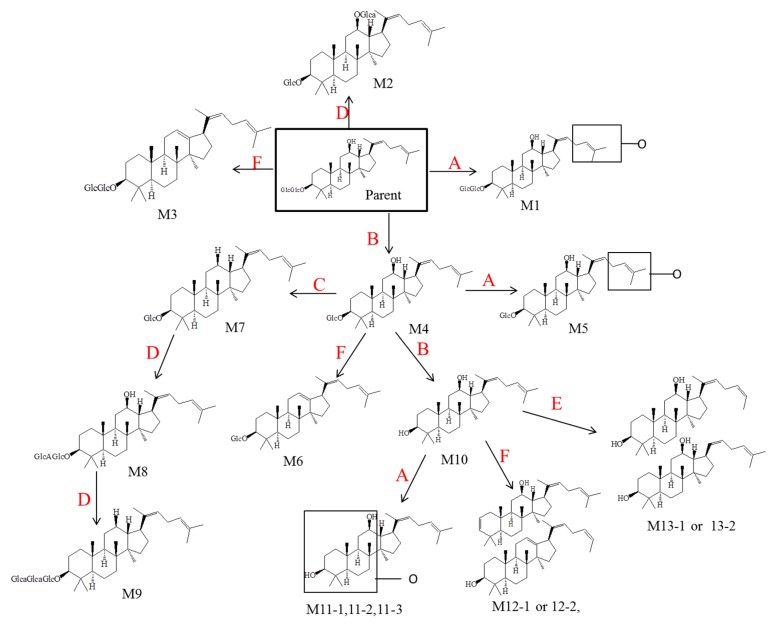
Major metabolic pathways proposed for ginsenoside Rg5. (A represents for oxidation, B deglycosylation, C deoxidation, D glucuronidation, E demethylation, F dehydration).

According to the results mentioned above, six metabolic pathways of Rg5 were proposed and depicted in Figure 6 to explain the formation of the 17 metabolites. Several reactions were included in these pathways, which are deglycosylation, oxidation, deoxidation, glucuronidation, demethylation and dehydration. Therefore, all of the metabolism products were involved in both phase I and phase II metabolism pathways. 

## 3. Materials and Methods

### 3.1. Materials

Ginsenoside Rg5 (purity > 99%) was purchased from the Shanghai Ginposome Pharmatech Co., Ltd. (Shanghai, China). Distilled deionized water was generated from Millipore Milli-Q Gradient system (Millipore, Bedford, MA, USA). Acetonitrile, methanol, formic acid of HPLC grade were purchased from Fisher Scientific Co. (Santa Clara, CA, USA). RLMs were obtained from our laboratory [40]. Magnesium chloride, tris base, alamethicin, hydrochloric acid, heparin sodium, nicotinamide adenine dinucleotide phosphate (NADPH), uridine diphosphate glucuronic acid (UDPGA), glucose-6-phosphate dehydrogenase, glucose 6-phosphate, were purchased from Sigma–Aldrich Co. (St. Louis, MI, USA).

### 3.2. In Vitro Microsomal Incubation with NADPH and UDPGA

In vitro phase I/II metabolism study was carried out by incubating Rg5 in RLMs with NADPH-generating system and UDPGA. Ethanol was used to prepare the stock solution of Rg5, and the final concentration of ethanol in the system was controlled to no more than 1% (*v*/*v*). Rg5 (500 μM), microsomes (1.5 mg protein/mL) and alamethicin (20 g/mg protein) were mixed together in 50 mM Tris–HCl buffer with a pH of 7.4 and incubated for 5 min at 37 °C. The incubation reaction were initiated with the addition of NADPH-generating system, which consisted of 1 mM NADPH, 5 mM UDPGA, 10 mM glucose 6-phosphate, 1 unit/mL of glucose 6-phosphate dehydrogenase and 4 mM magnesium chloride. After incubation for 1 h, the reaction was terminated with 800 μL ice-cold acetonitrile and centrifuged for 15 min (10,000× *g*). Then, 750 μL supernatant was evaporated with a gentle stream of N_2_ until dryness at 40 °C. The residue was reconstituted with 200 μL 95% methanol with vortexing. After centrifugation for 15 min at 10,000× *g*, 10 μL of the supernatant was taken to inject into the UPLC/QTOF-MS system. The samples without substrates, NADPH or UDPGA were prepared as controls. Each experiment was performed in triplicate.

### 3.3. Drug Administration and Sample Collection 

The metabolism process of Rg5 after oral administration in rats was studied by determining the original compound and metabolites in the urine, plasma and feces. Male Sprague–Dawley rats (220 ± 20 mg) were purchased from Shanghai SLAC Laboratory Animal Co. Ltd. (Shanghai, China). The rats were fasted for 12 h, but with access to water before the administration of Rg5. After the blank urine, plasma and feces samples were collected, an oral dose (50 mg/kg) of Rg5 in water containing 0.5% croscarmellose sodium (CMC-Na) was given to each rat by gastric intubation. The feces and urine were collected from 0 to 24 h using metabolic cages. To obtain plasma sample, 0.5 mL of blood sample was collected through the oculi chorioideae vein after 4 h of administration. After centrifugation at 3000× *g* for 10 min, plasma was obtained. All samples were stored at −80 °C before analysis.

### 3.4. Sample Preparation

To find the best UPLC/QTOF-MS analysis conditions, a sample of 5 μg/mL Rg5 solution (dissolved in 95% methanol) was prepared firstly. After centrifugation at 10,000× *g* for 15 min, 10 μL of the supernatant was analyzed by UPLC/QTOF-MS systems.

Urine samples: 1 mL urine sample was mixed with acetonitrile of same volume thoroughly. After centrifugation at 10,000× *g* for 15 min, supernatant (1.5 mL) was evaporated to dryness at 40 °C under a gentle stream of N_2_. Then 200 μL 95% methanol was added to reconstitute residue with vortexing. After centrifugation at 10,000× *g* for 15 min, 10 μL of the supernatant was applied to UPLC/QTOF-MS analysis. 

Feces samples: 1 g of each feces sample was suspended in 10 mL methanol by ultrasonication, Then 1 mL supernatant was dried and the residue was dissolved in 200 μL 95% methanol. After centrifugation for 15 min at 10,000× *g*, 10 μL of the supernatant was applied to the UPLC/QTOF-MS analysis. Plasma samples: 800 μL acetonitrile was added into 200 μL plasma and mixed by vortexing for 1 min. After centrifugation for 10 min at 4 °C (10,000× *g*), the supernatant was dried under a gentle stream of N_2_ (40 °C). The residue was dissolved by 50 μL 95% methanol. After centrifugation at 10,000× *g* for 15 min, supernatant (10 μL) was applied to the UPLC/QTOF-MS analysis.

### 3.5. UPLC Chromatography and Q/TOF Mass Spectrometer 

For separation, an Eclipse plus C_18_ reversed phase LC column (50 × 2.1 mm, i.d. 1.8 μm, Agilent, Santa Clara, CA, USA) was used for chromatographic analysis. Gradient elution was performed using the following mobile phase systems: 0.5% formic acid in deionized water (A) and acetonitrile (B); The gradient profile was as follows: held at 5% B for 0–1 min; increased 5–20% B linearly for 1–1.5 min; increased 20–70% B linearly for 1.5–6.5 min; increased 70–100% B linearly for 6.5–12 min; held at 100% B for 1 min and then decreased to 5% B to equilibrate the column. The sample-tray temperature was set at 4 °C, while the flow rate was 0.3 mL/min and the column temperature was kept at 30 °C. 

Q/TOF mass spectrometer was applied with an ESI source in positive ion mode (ion spray voltage: 5.5 kV, ion source temperature: 121 °C). Data acquisition was performed from 100 to 1000 Da with an accumulation time of 0.25 s set in positive full scan mode. The collision energy and declustering potential (DP) were set to 10 eV and 80 V, respectively. Nitrogen (including the heater gas, the nebulizer gas and the curtain gas) was set at 50, 50 and 30 psi, respectively. 

### 3.6. Data Analysis

The data analyses were performed using PeakView (v2.2) and MasterView (v1.2) programs (AB Sciex). Through comparing the mass spectrogram and retention time of an array of extracted ion chromatograms of experimental samples with the control ones, the common chromatographic peaks were eliminated and potential metabolites were detected. Based on the transformation of fragmentation patterns, mass shift and retention time, structures of the potential metabolites of Rg5 were determined.

## 4. Conclusions

A quick and reliable analytical tool has been established to investigate the metabolism of Rg5 in rats both in vitro and in vivo. Although the exact structures of metabolites in some caseses could not be unequivocally established using mass spectrometry alone, the present study provides crucial insights to better understand the metabolic pathways of Rg5. By investigating the metabolism of Rg5 and other related ginsenosides, useful information on the both efficacy and safety aspects could be provided as a reference for pharmacological research. Also, many more drug candidates and active compounds with drug potential can be optimized and identified based on the results in the study. Further pharmacokinetics data of Rg5 in vivo is still urgently needed to comprehensively understand the absorption, distribution and excretion behaviors of different metabolites in the organism.

## Figures and Tables

**Table 1:** Retention time (t_R_) and fragment ions of the metabolites of Rg5 in rat by UPLC/TOF-MS analysis.

No.	Formula	t_R_ (min)	Experimental *m*/*z*	ppm Error	Source	Pathway
Parent	C_42_H_70_O_12_	5.95	767.4914	−3	FP	1
M1	C_42_H_70_O_13_	11.31	783.4855	−4.4	P	1
M2	C_48_H_78_O_18_	4.3	943.5231	−3.1	U	2
M3	C_42_H_68_O_11_	12.05	749.4838	0.5	F	1
M4	C_36_H_60_O_7_	7.38	605.4374	−3.5	F, P, R	1
M5	C_36_H_60_O_8_	4.87	621.4351	4.87	R	1
M6	C_36_H_58_O_6_	7.38	587.4283	−3.9	F	1
M7	C_36_H_60_O_6_	11.2	589.446	−0.5	F	1
M8	C_42_H_68_O_12_	4.84	765.4757	−3.5	F, R	2
M9	C_48_H_76_O_18_	4.64	941.5082	−2.3	U	2
M10	C_30_H_50_O_2_	10.69	443.3886	0.5	F, R	1
M11-1	C_30_H_50_O_3_	8.2	459.3818	−3.2	F	1
M11-2	C_30_H_50_O_3_	6.33	459.3817	−3.5	F	1
M11-3	C_30_H_50_O_3_	9.01	459.3817	−3.4	F	1
M12-1	C_30_H_48_O	10.51	425.3768	−2.4	F,	1
M12-2	C_30_H_48_O	5.98	425.3771	−1.7	F,	1
M13-1	C_29_H_48_O_2_	10.54	429.3727	0	FP	1
M13-2	C_29_H_48_O_2_	9.89	429.3723	−1	FP	1

P represents the plasma samples, U the urine samples, F the feces samples and R the in vitro samples, I represents the phase I metabolic pathway and II represents the phase II metabolic pathway.
